# Resistance, Ineffectiveness, and Off-Label Use Related to Cephalosporins from the Reserve Group—A Pharmacovigilance Signal Detection Study on EudraVigilance Database

**DOI:** 10.3390/ph19010155

**Published:** 2026-01-15

**Authors:** Anca Maria Arseniu, Bogdan Ioan Vintila, Anca Butuca, Laurentiu Stoicescu, Adina Frum, Adriana Aurelia Chis, Rares Arseniu, Felicia Gabriela Gligor, Steliana Ghibu, Claudiu Morgovan, Carmen Maximiliana Dobrea

**Affiliations:** 1 Preclinical Department, Faculty of Medicine, “Lucian Blaga” University of Sibiu, 550169 Sibiu, Romania; anca.arseniu@ulbsibiu.ro (A.M.A.); adina.frum@ulbsibiu.ro (A.F.); felicia.gligor@ulbsibiu.ro (F.G.G.); claudiu.morgovan@ulbsibiu.ro (C.M.); carmen.dobrea@ulbsibiu.ro (C.M.D.); 2 Clinical Surgical Department, Faculty of Medicine, “Lucian Blaga” University of Sibiu, 550169 Sibiu, Romania; bogdan.vintila@ulbsibiu.ro; 3 Department of Cardiology, 5th Medical Clinic, Faculty of Medicine, “Iuliu Haţieganu” University of Medicine and Pharmacy, 400139 Cluj-Napoca, Romania; 4 Association for Excellence in Pharmaceutical Education and Research, 550169 Sibiu, Romania; a.adriana.chis@gmail.com; 5 Department of Clinical Medicine, Faculty of Medicine, “Lucian Blaga” University of Sibiu, 550169 Sibiu, Romania; rares.arseniu@ulbsibiu.ro; 6 Department of Pharmacology, Physiology and Pathophysiology, Faculty of Pharmacy, “Iuliu Haţieganu” University of Medicine and Pharmacy, 6A Louis Pasteur Street, 400349 Cluj-Napoca, Romania; stelianaghibu@yahoo.com

**Keywords:** cephalosporins, AWARE, EudraVigilance, antibiotic resistance, ineffectiveness, off-label use, disproportionality analysis, pharmacovigilance

## Abstract

**Background/Objectives**: Antimicrobial resistance (AMR) is considered a major threat by the healthcare community. In this context, the AWaRe (Access, Watch, Reserve) classification of antibiotics is a valuable tool that can assist physicians during the clinical decision process and pharmacists in promoting the rational use of antibiotics. Pharmacovigilance studies based on real-world evidence offer valuable insight into the AMR phenomenon. The aim of this study was the assessment of the resistance, ineffectiveness, and off-label use signals of all five cephalosporins belonging to the Reserve group (ceftazidime/avibactam, ceftaroline, cetolozane/tazobactam, ceftobiprole, and cefiderocol). **Methods**: The study was conducted using descriptive approaches on EudraVigilance data and disproportionality analyses comparing each of the fourteen cephalosporins in the Watch group. **Results**: Ceftazidime/avibactam (*n* = 904, 38.6%) topped the reports, followed by ceftaroline (*n* = 559, 23.9%) and ceftolazane/tazobactam (*n* = 560, 23.9%). The lowest number of reports was submitted for cefiderocol (*n* = 176, 7.5%) and ceftobiprole (*n* = 146, 6.2%). The resistance to ceftazidime/avibactam, cefiderocol, and ceftolozane/tazobactam was reported with a higher probability than all others, the strongest signal being observed for cefiderocol against cefixime (ROR: 171.25, 95% CI 79.64–368.27). All cephalosporins from the Reserve group (except ceftobiprole) have higher probability for reporting ineffectiveness than cephalosporins from the Watch group; the strongest signal was observed for cefiderocol–cefditoren (ROR: 14.70, 95% CI 6.73–32.11). All cephalosporines from the Reserve group had a higher probability of reporting off-label use by comparison with the ones from the Watch group, except for two cases of no disproportionate signal between cefiderocol–cefoperazone and cefiderocol–ceftizoxime; the strongest signal was observed for ceftolozane/tazobactam–cefotaxim (ROR: 43.61, 95% CI 30.14–63.09). **Conclusions**: This analysis supplements information from clinical trials and current clinical practice, underscoring the critical need for rigorous antibiotic stewardship programs. Notably, even restricted use of cephalosporins demonstrated therapeutic failure and inappropriate utilization.

## 1. Introduction

Microorganism resistance to antibiotics, also known as antibacterial resistance, together with resistance to antiviral, antifungal, and antiparasitic agents, is encompassed within the broader phenomenon of antimicrobial resistance (AMR), which has become a major concern for health systems, with numerous medical, social, and economic implications, being associated with increased morbidity, mortality, and healthcare costs [1,2]. The adaptation of microorganisms to the action of antibiotics (ABs) is a dynamic phenomenon, as they develop various resistance mechanisms that can often lead to a decrease in therapeutic efficacy, but also to the aggravation of pathologies or the appearance of complications, which can compromise the patient’s immune system and can sometimes even cause therapeutic ineffectiveness [3]. The emergence of multidrug-resistant bacteria (MDR) and extensively drug-resistant bacteria (XDR) leads to a decrease or even loss of AB efficacy and an increase in morbidity and mortality in general as previously manageable infections are becoming harder and sometimes impossible to treat [4,5].

The World Health Organization (WHO) warns that AMR is currently one of the top 10 threats to global health and estimates that across the globe, in 2019, about 1.27 million deaths were directly attributed to drug-resistant infections [1]. Moreover, infections generated by AMR microorganisms could increase by almost 70% by 2050—with more than 39 million cumulative deaths between 2025 and 2050 [6].

The excessive and inadequate consumption of Abs, both in the treatment of infections and in their use in food, animal, and plant production, has led to the continuous increase in microbial resistance, to which is added the fact that the development of new antimicrobial agents takes a long time to be introduced into current therapy [7]. The misuse of Abs and insufficient prevention, together with insufficient infection control in hospitals, inadequate sanitation, and improper food handling, are the main factors underlying the development of AMR [8].

In recent years, there have been increasing global concerns about developing new strategies to limit or even combat AMR. To prevent the occurrence of AMR and MDR, it has become necessary to design successful infection prevention strategies and effective approaches to the treatment of an increasing range of infections caused by resistant bacteria, but also to highlight the impact of the development of resistant microorganisms on health and society in general [9]. Thus, along with the implementation of AB stewardship programs in healthcare environments to promote the rational use of ABs, the aim is to strengthen infection prevention and control strategies in hospitals and other units, which would limit the dissemination of resistant microorganisms. Improving hygiene, active screening for high-risk patients, isolating patients with resistant infections, and decontaminating surfaces and equipment, along with continuous information on side effects and risks due to the emergence of AMR, are effective strategies in this direction [10]. Moreover, to monitor antibiotic use, to support effective antimicrobial stewardship, to facilitate efficient treatment, and to reduce AMR, the WHO has introduced the AWaRe classification of ABs, primarily encouraging the use of ABs from the Access group and recommending careful monitoring for the Watch group. The Reserve group is considered the last resort [11]. Antibiotics included in the Access and Watch groups are intended to treat common infections in the community or hospital settings, while antibiotics in the Reserve group are intended to treat infections caused by multidrug-resistant bacteria [12].

New cephalosporins with activity against multidrug-resistant pathogens, such as ceftaroline, ceftolozane/tazobactam, ceftazidime/avibactam, cefiderocol, and ceftobiprole, have been introduced in therapy [13,14,15,16,17]. Due to their favorable risk–benefit profile and lack of treatment alternatives, they have all been included in the WHO category of Reserve ABs [18].

Ceftaroline fosamil was approved in 2010 for the treatment of adult patients with (i) acute bacterial skin and soft tissue infections with resistant Gram-positive and common Gram-negative bacteria and (ii) community-acquired bacterial pneumonia. It is active on methicillin-resistant *Staphylococcus aureus* (MRSA) and multidrug-resistant *Streptococcus pneumoniae*, but it is not active against *Pseudomonas aeruginosa*, *Bacteroides fragilis*, and carbapenem-resistant Enterobacteriaceae. It is used off-label for osteomyelitis, endocarditis, and meningitis [19,20,21,22,23].

Ceftolozane/tazobactam is a new combination antibiotic that consists of a strong antipseudomonal cephalosporin, ceftolozane, and a β-lactamase inhibitor, tazobactam. Ceftazidime/avibactam is the combination of a third-generation cephalosporin, ceftazidime, and a non-β-lactam β-lactamase inhibitor, avibactam. Both combinations are intravenously administered for the treatment of complicated intra-abdominal infections, complicated urinary tract infections, hospital-acquired bacterial pneumonia, and ventilator-associated bacterial pneumonia [14,24,25]. Off-label uses of ceftolozane/tazobactam include skin and soft-tissue infections, bone and joint infections, and bloodstream infections [26].

Of interest is the development of new ABs that have different mechanisms of action than the classic ones, such as cefiderocol, a new cephalosporin substituted by catechol, structurally similar to cefepime and ceftazidime. In addition to passive diffusion through the porin-like channels of the outer membrane, cefiderocol is a siderophore, undergoing active transport in the bacterial cell through the iron channels of Gram-negative bacteria, with in vitro proven efficiency against strains resistant to several drugs, including strains producing broad-spectrum β-lactamase and carbapenemase [27].

Ceftobiprole is a fifth-generation cephalosporin overcoming the limitations of previous generations, as it is effective both against Gram-positive and Gram-negative pathogens, including multidrug-resistant ones. Compared to other drugs used to treat MRSA infections, ceftobiprole exhibits better antibacterial activity, reduced toxicity, and improved tolerability [28].

Off-label use can present certain risks because the safety and efficacy of the drug have not been demonstrated for the intended indication. Potential risks include unknown side effects; drug interactions; reduced therapeutic effectiveness; and, in the case of antibiotics, off-label use may also contribute to the development of bacterial resistance [29].

Pharmacovigilance is the branch of pharmaceutical sciences dedicated to continuous monitoring and updating the safety profiles of medicines. Established databases such as EudraVigilance (EV), developed by the European Medicines Agency, are used for the reporting and evaluation of adverse drug reactions (ADRs) [30]. For medicines such as the Reserve-group ABs, prescribed exclusively in severe infections, real-world studies bring valuable information that, in addition to clinical studies data, contribute to generating a comprehensive safety profile.

The aim of this study was the assessment of the resistance, ineffectiveness, and off-label use of the five cephalosporins belonging to the Reserve group, based on real-world evidence from the EV database, by descriptive and disproportionality approaches.

## 2. Results

### 2.1. General Characteristics Presented in ICSRs

**Figure 1:**
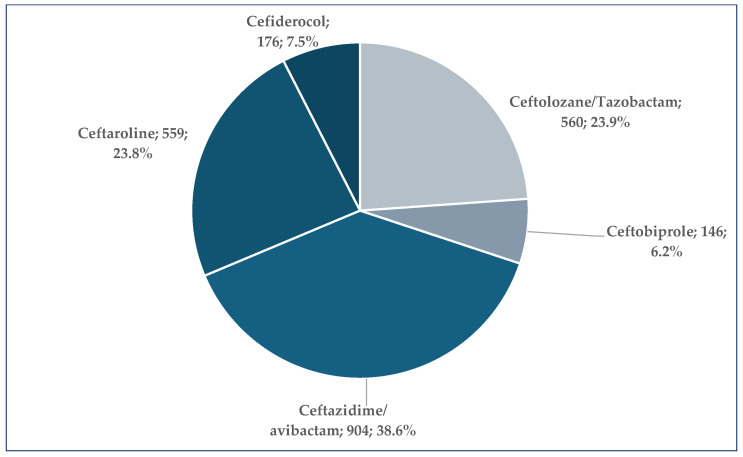
Distribution of ICSRs by cephalosporins included in Reserve group.

Five cephalosporins from the Reserve group have been included in this study: (i) the third-generation ceftazidime/avibactam; (ii) the fifth-generation ceftaroline, cetolozane/tazobactam, and ceftobiprole; and (iii) the siderophore cephalosporin cefiderocol. Most ICSRs were registered for ceftazidime/avibactam (*n* = 904, 38.6%). For ceftaroline (*n* = 559, 23.9%) and ceftolazane/tazobactam (*n* = 560, 23.9%), a similar number of ICSRs have been submitted in the EV database. The lowest number of ICSRs was registered for cefiderocol (*n* = 176, 7.5%) and ceftobiprole (*n* = 146, 6.2%) (Figure 1).

A higher frequency of cases was registered for patients included in the 18–64 years group. In this group, ICSRs related to ceftobiprole (32.9%) and ceftolozane/tazobactam (32.7%) were reported with the lowest frequency, while the highest frequency was noticed for cefiderocol (48.9%). In the 65–85 years group, the highest frequency of cases was related to ceftobiprole (48.6%), and the lowest for ceftolozane/tazobactam (29.3%). For all drugs of interest, ADRs were reported more frequently for males than for women (ceftobiprole (54.1%) and ceftazidime/tazobactam (59.6%)). Reports from the European Economic Area (EEA) have been more frequent for cefiderocol (81.3%), ceftobiprole (80.8%), and ceftalozane/tazobactam (55.7%). On the other hand, ADRs reported for ceftaroline and ceftazidime/avibactam in non-EEA countries have been more frequent than in EEA countries. The majority of reports were filled by healthcare professionals (HPs). The minimum percentage of ICSRs filled by HPs was observed for ceftazidime/avibactam (87.7%) (Table 1).

### 2.2. Descriptive Analysis of Reported ADRs

The highest frequency of reports by SOC was observed in the “General disorders and administration site conditions” and “Injury, poisoning, and procedural complications” SOCs. High frequencies were registered in the “Infections and infestations” SOC (except ceftobiprole—5.5%). Also, even though the frequencies of ADRs in each SOC are comparable for all five cephalosporins, some differences could be observed. For example, for “Blood and lymphatic disorders”, the highest frequency was reported for ceftaroline (38.3%), a value greater than for the other cephalosporins. Compared to cefiderocol (10.2%) and ceftazidime/avibactam (8.1%), a lower incidence was noticed for “Hepatobiliary disorders” related to ceftaroline (2.5%), ceftobiprole (3.4%), and ceftolozane/tazobactam (3.8%). Opposite to ceftobiprole (37.0%), the frequency of “Neurological disorders” was lower for ceftaroline (7.2%) and ceftolozane/tazobactam (8.6%). Also, “Renal and urinary disorders” had a lower incidence for ceftobiprole (4.8%) and ceftaroline (7.7%) compared to the other three cephalosporines, but “Skin and subcutaneous tissue disorders” registered a much higher frequency than for the others (ceftaroline—19.3% and ceftobiprole—26.0%) (Supplementary Materials Table S1).

**Figure 2:**
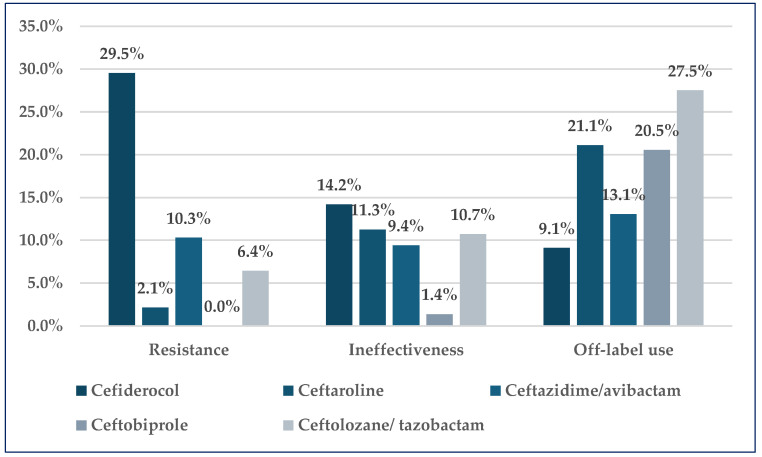
The frequency of ADRs related to resistance, ineffectiveness, and off-label use in total reports.

Cefiderocol, which is a siderophore cephalosporin, presents the highest rate of resistance (29.5%) and ineffectiveness (14.2%) in total reports, and the lowest rate of off-label use (9.1%). ADRs related to the resistance of ceftazidime/avibactam represented 10.3% of the total. The rate of off-label use of ceftolozane/avibactam is the highest of all five Reserve cephalosporins (27.5%). The ineffectiveness of ceftaroline (11.3%), ceftazidime/avibactam (9.4%), and ceftolozane/avibactam (10.7%) has similar rates. On the other hand, the reports related to ceftobiprole resistance and ineffectiveness were the lowest (0.0% and 1.4%), but off-label use registered a high rate (20.5%). Similarly, a high rate of off-label use was noticed for ceftaroline (21.1%) (Figure 2).

**Figure 3:**
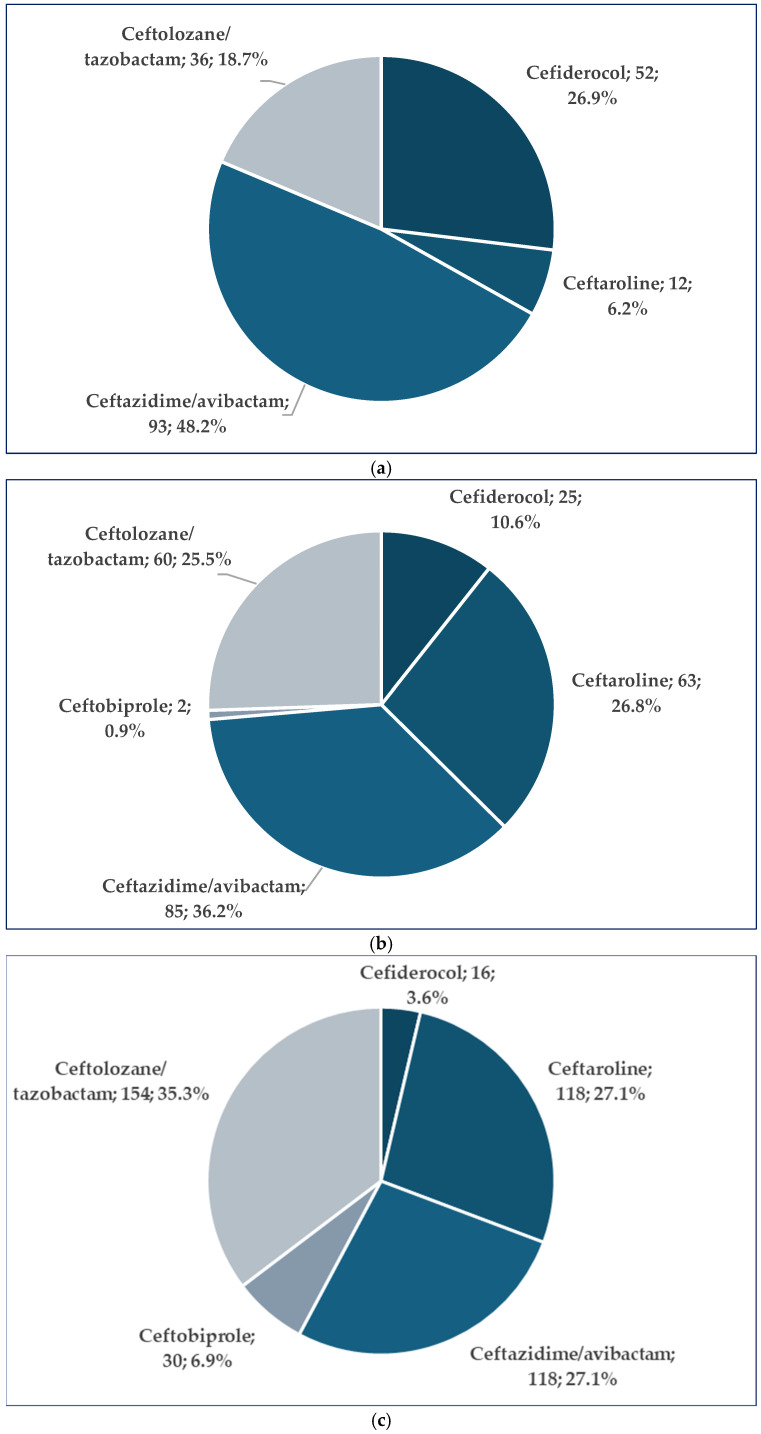
Distribution of ADRs. (**a**) ADRs related to resistance; (**b**) ADRs related to ineffectiveness; (**c**) ADRs related to off-label use.

According to Figure 3a, ceftazidime/avibactam has the highest number of ADRs related to resistance (*n* = 93, 48.2%), followed by cefiderocol (*n* = 52, 26.9%). No reports related to resistance were submitted in the EV database for ceftobiprole. Ineffectiveness was reported to be more frequent for ceftazidime/avibactam (*n* = 85, 36.2%), ceftaroline (*n* = 63, 26.8%), and ceftolozane/tazobactam (*n* = 60, 25.5%) (Figure 3b). Off-label use was more frequently reported for ceftolozane/tazobactam (*n* = 154, 35.3%). For ceftazidime/avibactam and ceftaroline, the same number of ADRs was registered (*n* = 118, 27.1%) (Figure 3c).

**Figure 4:**
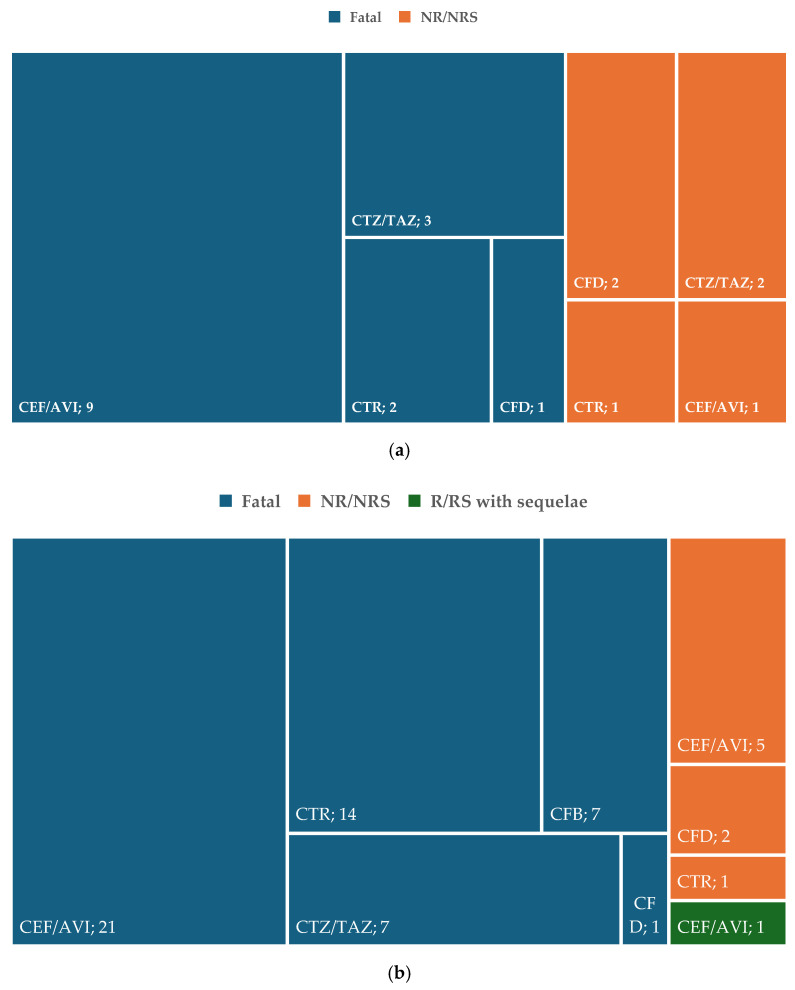

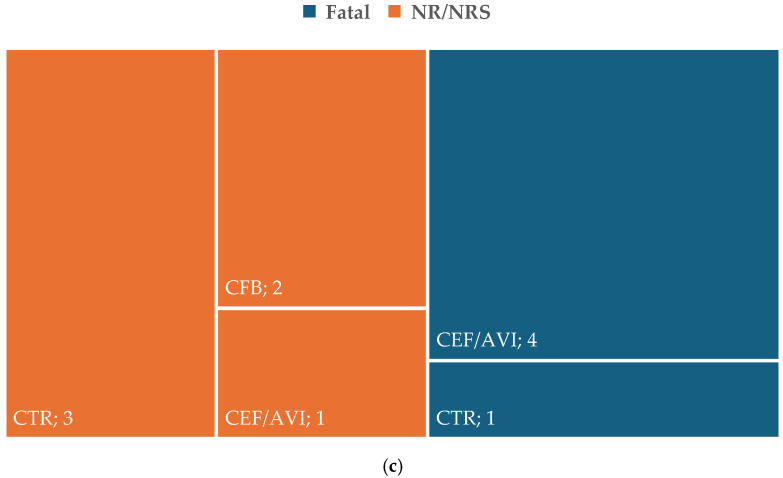
Structure of ADRs with unfavorable outcomes. (**a**) ADRs related to resistance; (**b**) ADRs related to ineffectiveness; (**c**) ADRs related to off-label use. CEF/AVI—ceftazidime/avibactam; CFB—ceftobiprole; CFD—cefiderocol; CTR—ceftaroline; CTZ/TAZ—ceftolozane/tazobactam.

Figure 4a presents the distribution of ADRs related to resistance with unfavorable outcomes. No unfavorable outcomes have been reported for ceftobiprole resistance. For ADRs related to drug resistance, fifteen cases were fatal (nine—ceftazidime/avibactam, three—ceftolozane/tazobactam, two—ceftaroline, and one—cefiderocol) and six cases were not recovered or not resolved (cefiderocol—two, ceftolozane/tazobactam—two, ceftaroline—one, and ceftazidime/avibactam—one). In Figure 4b, the distribution of ADRs related to ineffectiveness with unfavorable outcomes is represented. In total, 50 cases were fatal (21 were related to ceftazidime/avibactam, 14—ceftaroline and 7—ceftobiprole, respectively, ceftolozane/tazobactam and 1—cefiderocol). A single case was resolved or recovered with sequelae (ceftazidime/avibactam) and eight cases were not recovered or resolved (cefiderocol—two, ceftaroline—one, and ceftazidime/avibactam—five). On the other hand, off-label use led to death in five cases (four—ceftazidime/avibactam and one—ceftaroline) and six cases were not recovered or resolved (ceftaroline—three, ceftazidime/avibactam—one, and ceftobiprole—two) (Figure 4c).

### 2.3. Disproportionality Analysis

#### 2.3.1. Signals Related to Drug Resistance

**Figure 5:**
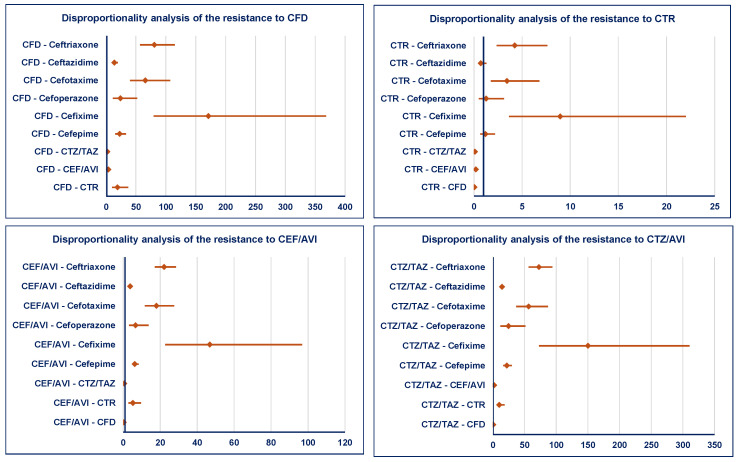
Disproportionality analysis of ADRs related to resistance of cephalosporins from the Reserve group. CEF/AVI—ceftazidime/avibactam; CFD—cefiderocol; CTR—ceftaroline; CTZ/TAZ—ceftolozane/tazobactam.

No ADRs of resistance to ceftobiprole have been reported. By comparison with the other cephalosporins from the Watch group, the resistance to ceftazidime/avibactam, cefiderocol, and ceftolozane/tazobactam was reported with a higher probability than all others. Regarding the resistance to ceftaroline, a higher probability of reporting was observed only by comparison with cefixime, cefotaxime, and ceftriaxone. Resistance to cefiderocol is more probable to be reported compared to all other cephalosporins from the Reserve group. Ceftaroline presents a lower risk of reporting resistance (Figure 5).

#### 2.3.2. Signals Related to Ineffectiveness

**Figure 6:**
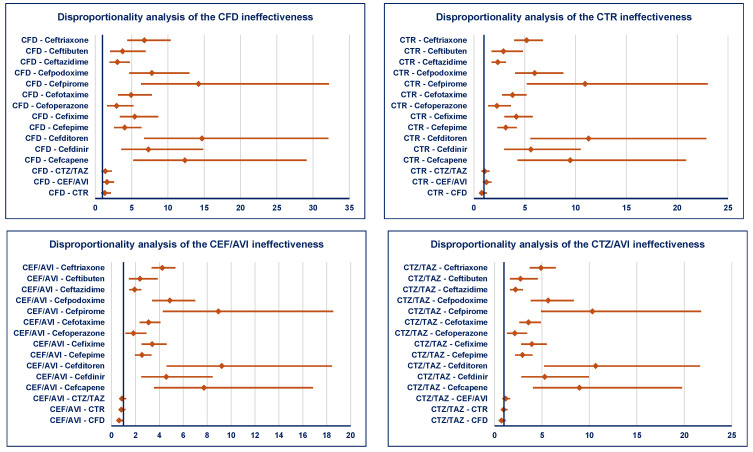
Disproportionality analysis of ADRs related to ineffectiveness of cephalosporins from the Reserve group. CEF/AVI—ceftazidime/avibactam; CFD—cefiderocol; CTR—ceftaroline; CTZ/TAZ—ceftolozane/tazobactam.

Only two ADRs related to the ineffectiveness of ceftobiprole have been identified. According to EMA recommendations (number of signals must be a minimum of five), for ceftobiprole, it could not be established whether the signal is disproportionate or not. All cephalosporins from the Reserve group (except ceftobiprole) have a higher probability of reporting ineffectiveness than cephalosporins from the Watch group. Regarding the comparison between drugs within the Reserve group, no disproportionate signal was observed (Figure 6).

#### 2.3.3. Signals Related to Off-Label Use

**Figure 7:**
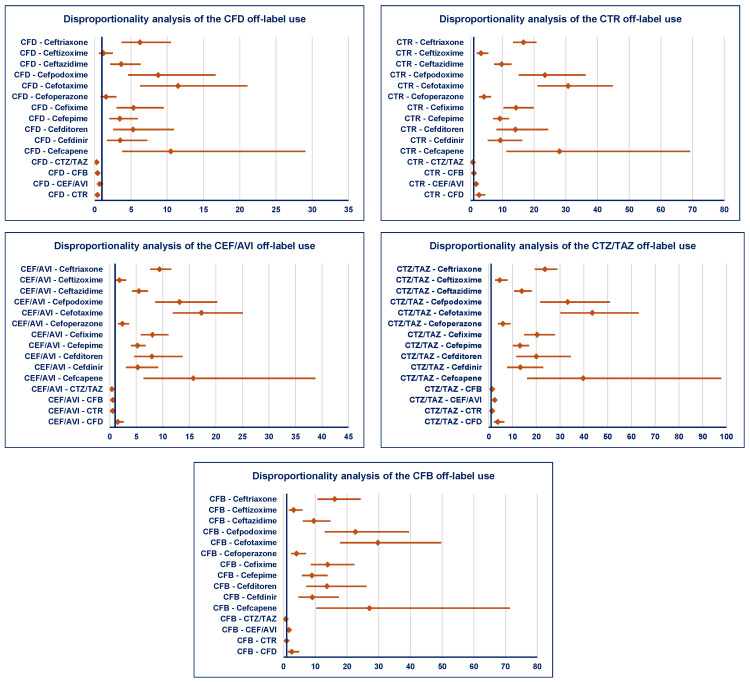
Disproportionality analysis of ADRs related to off-label use of cephalosporins from the Reserve group. CEF/AVI—ceftazidime/avibactam; CFB—ceftobiprole; CFD—cefiderocol; CTR—ceftaroline; CTZ/TAZ—ceftolozane/tazobactam.

Regarding the disproportionality in the Reserve group, a lower probability of reporting off-label use was observed for ceftazidime/avibactam by comparison to the following: (i) ceftaroline (ROR: 0.56, 95% CI: 0.42–0.74); (ii) ceftobiprole (ROR: 0.58, 95% CI: 0.37–0.91); and (iii) ceftolozane/tazobactam (ROR: 0.40, 95% CI: 0.30–0.52). No differences were registered between the following: cefiderocol and ceftazidime/avibactam; ceftaroline and ceftobiprole; and ceftolozane/tazobactam and ceftobiprole. All cephalosporines from the Reserve group had a higher probability of reporting off-label use by comparison with the others from the Watch group, except two cases of no disproportionate signal between cefiderocol–cefoperazone and cefiderocol–ceftizoxime (Figure 7).

The outcomes of this analysis reflect population-level reporting trends related to suspected resistance, lack of effectiveness, and the off-label use of Reserve-group cephalosporins, rather than verified treatment failure. The results represent patterns of disproportionate reporting of resistance, ineffectiveness, and off-label use among cephalosporins, rather than confirmed clinical diagnoses or therapeutic failures. The spontaneous nature of the reporting system limited the availability of comprehensive clinical details. Key information, such as dosage, type and characteristics of infection, and concurrent therapies, was often absent, preventing in-depth clinical stratification at the individual patient level.

## 3. Discussion

EudraVigilance, the EU database for collecting and analyzing suspected ADRs, ensures drug safety and public health by rapid detection and evaluation of new and changing safety issues [31]. The emergence of AMR requires multidisciplinary approaches, and pharmacovigilance can strengthen current antimicrobial use strategies [32]. In addition to current strategies for monitoring AMR, pharmacovigilance databases containing spontaneous reports of suspected ADRs can serve as a source of data indicating the inappropriate use of antimicrobials and potential resistance [33].

The present study examined the safety profile, focusing on the resistance patterns, reported inefficacy, and off-label use of five cephalosporins classified as part of the Reserve group: (i) the third-generation ceftazidime/avibactam; (ii) the fifth-generation ceftaroline, ceftobiprole, and ceftolozane/tazobactam; and (iii) the siderophore cephalosporin cefiderocol. Using a descriptive approach, we evaluated their safety profiles based on safety reports uploaded in the EV database. Additionally, disproportionality analyses facilitated comparisons within the analyzed cephalosporins from the Reserve group and against other cephalosporins from the Watch group, providing insights into their relative safety signals and usage trends in real-world settings.

Among the cephalosporins in the Reserve group, ceftazidime/avibactam accounted for the highest number of ICSRs (*n* = 904, 38.6%). On the other hand, the lowest numbers of ICSRs were reported for cefiderocol (*n* = 176, 7.5%) and ceftobiprole (*n* = 146, 6.2%). The high number of reports related to ceftazidime/avibactam may be influenced by several factors, such as the availability of the drug on the market for a longer time, a broader spectrum increasing its use, prescription trends, the capacity of microorganisms to adapt and develop resistance mechanisms, and the condition of the patient itself. In clinical settings, Gram-negative infections unresponsive to other antibiotics are becoming more widespread, including those caused by carbapenem-resistant Enterobacteriaceae [34,35,36,37], which is triggering an increase in the prescription rate of ceftazidime/avibactam. Furthermore, increased resistance to ceftazidime/avibactam has been reported, especially for carbapenem-resistant Gram-negative bacteria, which may reach 18% for *Pseudomonas aeruginosa* and exceed 50% for *Acinetobacter baumannii* [38,39], thus making clinicians more aware of the safety and efficacy of this drug. Regarding the condition of the patients, the polypharmacy often required to treat critically ill patients and a poor health state itself, due to multiple comorbidities, increase the risk of ADRs [40,41,42,43]. Pharmacists, key professionals in the healthcare system, have a crucial role regarding not only ABs, but all medicines, starting from the development phase to the post-marketing stage. Specifically, for controlling and limiting the negative impact of antibacterial resistance, pharmacists are involved in stewardship programs, optimization of AB use, monitoring of safety and effectiveness, and the prevention of inappropriate off-label use [1,2,3,4,5,6].

Our analysis revealed that individuals aged 18 to 64 accounted for the highest number of ADR reports related to cephalosporins in the Reserve group. This likely reflects the increased clinical exposure among working-age adults, who tend to have higher hospitalization rates and more frequent interactions with healthcare services [44]. Within this age group, cefiderocol had the highest reporting rate (48.9%), while ceftobiprole and ceftolozane/tazobactam were reported less often. In contrast, among individuals aged 65 to 85, ceftobiprole was the most commonly reported drug, with a reporting rate of 48.6%. This preference may be due to its wider use in elderly patients with specific infections, such as community-acquired pneumonia or hospital acquired pneumonia [45,46].

For all five cephalosporins in the Reserve group, ADRs were reported more commonly in males than females. The highest reporting rates were observed for ceftazidime/avibactam (59.6% male patients) and for cefiderocol (57.4% male patients). Even though, from this perspective, our findings align with other studies suggesting that men may experience more antibiotic-related ADRs [47,48], further investigations are needed to clarify the factors contributing to this disparity.

Noticeable variations were observed regarding the geographical origin of the reports. The reports from the EEA were more numerous for most of the studied drugs, namely, cefiderocol (81.3%), ceftobiprole (80.8%), and ceftolozane/tazobactam (55.7%), but less than 50% for ceftaroline (38.1%) and ceftazidime/avibactam (44.1%). The situation may be triggered by differences regarding the moment authorization is granted and the availability or variations in health policies after approval on different markets. For example, ceftaroline was approved in the USA in 2010 and two years later in the EU [22]. A shorter time interval was noted for ceftazidime/avibactam, approved by the FDA in 2015 [49] and one year later by the EMA [50]. On the other hand, in the case of cefiderocol, although the trend of being first approved in the US is maintained, the gap is shorter, less than six months between November 2019, when it was first approved in the US [51] and April 2020, when it was first approved in the EU [52]. Data interpretation on cefiderocol should also consider the fact that the drug is a relatively new member of the therapeutic arsenal, compared with other cephalosporins. The low frequency of reports related to ceftaroline and ceftazidime/avibactam originating within the EEA is consistent with results reported by several studies that mention other more widely used or more established antibiotics in this region [53,54,55].

Most ICSRs for all agents were submitted by HPs, indicating strong involvement in pharmacovigilance. Even for ceftazidime/avibactam, which had the lowest reporting rate from healthcare providers among the studied cephalosporins, submissions from HPs still represented 87.7% of all cases. This high level of engagement from healthcare providers strengthens the reliability of the data and highlights the significance of clinical reporting in monitoring the safety of antibiotics from the Reserve group [56,57]. Moreover, raising awareness about MedDRA terms associated with drug resistance among both healthcare and non-healthcare professionals could help increase the number of adverse drug reaction reports [32].

For the five Reserve cephalosporins studied, the most frequently observed SOCs were “General disorders and administration site conditions” and “Injury, poisoning, and procedural complications”. Except for ceftobiprole, the other four drugs were associated with many reports under the “Infections and infestations” SOC. The above-mentioned SOCs were also the most frequently observed in a post-marketing safety study of cefiderocol based on FAERS reports [58]. Similarly, an elevated number of ICSRs in the “Infections and infestations” class was reported for other broad-spectrum antibiotics such as carbapenems [59].

The “General disorders and administration site conditions” SOC include PTs related to drug resistance or ineffectiveness. On the other hand, PTs related to the off-label use are included in the “Injury, poisoning, and procedural complications” SOC.

Our analysis highlights notable differences in the resistance, ineffectiveness, and off-label use reports among Reserve-group cephalosporins. Cefiderocol, despite its complex mechanism of action, exhibited the highest rates of resistance (29.5%) and ineffectiveness (14.2%) of all reports from EV. This means that almost one in three adverse reactions are related to resistance and one in seven to ineffectiveness. The observed resistance can be attributed to several factors, taking into consideration the resilience and virulence of these strains in addition to the microorganism-related resistance mechanisms, the patient related factors include immune deficiency. Also, these values might be triggered by prescription patterns, supported by regulatory guidance strictly for MDR or XDR infections. To overcome these drawbacks in clinical practice, careful patient selection, optimized dosing strategies, and the potential use of combination therapies when administering cefiderocol should be considered [60,61,62,63]. Ceftolozane/tazobactam had the highest off-label use rate at 27.5%, indicating its expanding use beyond approved indications, especially in soft-tissue and bone infections [26].

The analysis of ADR distribution by category (Figure 3) reveals distinct patterns in Reserve-group cephalosporins’ clinical use and safety profiles. Ceftazidime/avibactam had the highest number of resistance-related cases, with 93 reports (48.2%), followed by cefiderocol, with 52 cases (26.9%). These findings highlight the use of these drugs in high-risk, multidrug-resistant infections and the associated risk of therapeutic failure. In contrast, no resistance-related ADRs were reported for ceftobiprole. This may be attributed to its more limited or targeted use, along with a lower potential for selecting resistance [60,64,65,66,67,68]. Regarding ineffectiveness, ceftazidime/avibactam had the highest number of reports, with a total of 85 (36.2%), followed by ceftaroline (63 reports, 26.8%) and ceftolozane/tazobactam (60 reports, 25.5%). This indicates similar challenges among these agents in achieving the desired clinical outcomes [40,69,70].

Regarding off-label use, ceftolozane/tazobactam had the highest number of reports (154 reports, 35.3%), followed by ceftazidime/avibactam and ceftaroline with the same number of reports (118 reports, 27.1%). Off-label uses of ceftolozane/tazobactam have been reported as a last resort option in severe cases when other treatments are not efficient (e.g., meningitis caused by extensively drug-resistant *Pseudomonas aeruginosa*) [71]. Also, ceftaroline is frequently used off-label due to its broad spectrum of activity and safety profile [72].

The analysis of ADRs with unfavorable outcomes highlights significant safety concerns associated with the resistance, ineffectiveness, and off-label use of Reserve-group cephalosporins (Figure 4). Resistance-related fatalities were linked most to ceftazidime/avibactam (*n* = 9), followed by ceftolozane/tazobactam (*n* = 3). In terms of ineffectiveness-related ADRs, fatal outcomes were most prevalent in the case of ceftazidime/avibactam (*n* = 21), followed by ceftaroline (*n* = 14). These figures reflect the potential for treatment failure in critically ill patients [41]. Off-label use of these medications was associated with five deaths, primarily involving ceftazidime/avibactam (*n* = 4) and ceftaroline (*n* = 1). These findings underscore the importance of careful clinical decision-making when prescribing these agents, particularly in the case of off-label use or for resistant infections or other situations when ineffectiveness may occur, as patient outcomes may be significantly compromised [40,73].

The results from the disproportionality analysis showed that higher reporting odds of drug resistance were found for ceftazidime/avibactam, cefiderocol, and ceftolozane/tazobactam, when compared to cephalosporins from the Watch group, and for cefiderocol when compared to all other cephalosporins from the Reserve group. On the contrary, ceftaroline presents a lower risk of reporting drug resistance.

All analyzed cephalosporins from the Reserve group, except ceftobiprole, have a higher probability of reporting ineffectiveness compared to cephalosporins from the Watch group. Between the analyzed drugs from the Reserve group, no disproportionate signals were observed.

Cephalosporines from the Reserve group had a higher likelihood of reporting off-label use by comparison with cephalosporins from the Watch group. On the contrary, a lower likelihood of reporting off-label use was observed for ceftazidime/avibactam by comparison to the following: (i) ceftaroline (ROR: 0.56, 95% CI: 0.42–0.74); (ii) ceftobiprole (ROR: 0.58, 95% CI: 0.37–0.91); and (iii) ceftolozane/tazobactam (ROR: 0.40, 95% CI: 0.30–0.52). Compared to cephalosporins in the Watch group, the likelihood of reporting off-label use all was higher for all analyzed cephalosporins from the Reserve group.

The disproportionality methods are extensively validated for the identification of potential safety and effectiveness concerns in real-world settings [7,8,9,10]. From a clinical point of view, the detected signals may indicate difficulties associated with the utilization of last-line cephalosporins, such as suboptimal or non-approved use and the possible development of resistance. Although these findings cannot be interpreted as evidence of a causal relationship, they may influence antibiotic stewardship strategies and indicate the direction for prioritizing clinical and microbiological investigations.

### Limitations of the Study

The findings of this study may be subject to significant bias as it lacks a denominator and the underreporting of ADRs is a common phenomenon. Furthermore, EV relies on voluntary and spontaneous reporting, allowing not only healthcare professionals but also non-healthcare professionals, such as patients or pharmaceutical companies, to submit reports. Also, the quality of documentation may differ between reports, and some reports contain gaps in patient-related information, such as age or gender information and medical history, etc., thus obstructing individual-level clinical assessment and comprehensive evaluation of patient-specific risk factors. In addition, the association between drugs and ADRs may be influenced by comorbidities, concomitant medications, and drug–drug interactions; therefore, any causal relationship between drugs and adverse drug reactions cannot be established based on data from the EV database alone and further evaluation through prospective studies is warranted.

## 4. Materials and Methods

### 4.1. Study Design

This study was designed as a retrospective pharmacovigilance analysis of reports on ineffectiveness, resistance, and off-label use uploaded in the EudraVigilance database for the following cephalosporins included in the Reserve group: ceftazidime/avibactam, ceftaroline, cetolozane/tazobactam, ceftobiprole, and cefiderocol. Aggregated data extracted from all Individual Case Safety Reports (ICSRs) registered on the https://www.adrreports.eu/ portal until 26 January 2025 (accessed on 29 January 2025) were analyzed [74]. ICSRs were issued by healthcare professionals or non-healthcare professionals from the EEA or non-EEA [75]. ICSRs do not contain patients’ personal information, and no ethics committee approval is required [76]. This research relied exclusively on open-access EV datasets. There was no use or review of personal medical records, so the definition of standard patient-level inclusion or exclusion criteria could not be applied.

### 4.2. EV Database and Selection Criteria

Inclusion criteria consisted of ICSRs listing one of the investigated cephalosporins as the suspected drug, on one hand, and on the other, containing the selected preferred terms related to resistance, ineffectiveness, or off-label use. Exclusion criteria focused on the absence of the drugs of interest. EV conducts periodic deduplication as part of data management activities [11].

For each of the five cephalosporins, all ICSRs containing pharmacovigilance signals submitted to EV were considered. According to the Medical Dictionary for Regulatory Activities (MedDRA), ADRs are codified in more than 25,000 preferred terms (PTs). Thus, the following PTs were considered for drug ineffectiveness, drug resistance, and off-label use (Supplementary Materials Table S2) [30,32,77].

### 4.3. Data Analysis

A descriptive analysis of the general characteristics (patients’ age, sex, geographical origin, and the category of reporters) of the ICSRs submitted for all five cephalosporins was performed. Subsequently, all ICSRs related to resistance (3 PTs), drug ineffectiveness (11 PTs), and off-label use (8 PTs) were considered and the frequency of cases with unfavorable outcomes related to these medical conditions was identified. The distribution by outcome of ADRs associated with all five cephalosporins was performed. The following different terms were used to present the clinical outcomes: (i) unfavorable outcome (“Fatal”; “Not recovered/Not resolved”); (ii) favorable outcome (“Recovered/Resolved”; “Recovering/Resolving”); and (iii) unknown outcome (“Unknown”) [77].

Moreover, to evaluate the probability of reporting drug ineffectiveness, drug resistance, or off-label use occurrence with these cephalosporins, a disproportionality analysis was performed. A signal could be considered disproportionated if the number of ICSRs was minimum 5 and the 95% CI of the reporting odds ratio (ROR) was greater than 1.0 [78,79,80].

The ROR could be calculated (Supplementary Materials Table S3) by comparison with other drugs used in common therapeutic areas and similar clinical contexts. In the present study, the comparison was performed using reports of all cephalosporins included in the Watch group (Table 2) and among the cephalosporins within the Reserve group.

## 5. Conclusions

As AMR remains a threat that continuously evolves, pharmacovigilance studies contribute to creating a comprehensive understanding of this phenomenon. This study identified disproportionate reporting signals of resistance, ineffectiveness, and off-label use among cephalosporins from the Reserve group compared to those from the Watch group. In general, compared to the fourteen cephalosporins in the Watch group, the five cephalosporins in the Reserve group have a higher probability of reporting ineffectiveness, drug resistance, and off-label use. Among the Reserve group, for drug resistance only, ceftobiprole had no reports and for ineffectiveness; it had only two reports. Although the study does not allow for individual-level clinical assessment or causal inference, the findings highlight relevant real-world reporting patterns that may reflect prescribing challenges and potential emerging resistance. This analysis supplements information from clinical trials and current clinical practice, underscoring the critical need for rigorous antibiotic stewardship programs. Notably, even restricted use of cephalosporins demonstrated therapeutic failure and inappropriate utilization.

## Figures and Tables

**Table 1:** General characteristic of ICSRs. EEA—European Economic Area; HPs—healthcare professionals; NS—not specified.

	Cefiderocol	Ceftaroline	Ceftazidime/Avibactam	Ceftobiprole	Ceftolozane/Tazobactam
	*n* (%)	*n* (%)	*n* (%)	*n* (%)	*n* (%)
Age category					
NS	21	107	155	13	169
	(11.9%)	(19.1%)	(17.1%)	(8.9%)	(30.2%)
0–1 Month	0	1	2	0	0
	(0.0%)	(0.2%)	(0.2%)	(0.0%)	(0.0%)
2 Months–2 Years	0	4	15	0	3
	(0.0%)	(0.7%)	(1.7%)	(0.0%)	(0.5%)
3–11 Years	1	5	13	0	6
	(0.6%)	(0.9%)	(1.4%)	(0.0%)	(1.1%)
12–17 Years	6	18	15	0	10
	(3.4%)	(3.2%)	(1.7%)	(0.0%)	(1.8%)
18–64 Years	86	233	348	48	183
	(48.9%)	(41.7%)	(38.5%)	(32.9%)	(32.7%)
65–85 Years	58	163	279	71	164
	(33.0%)	(29.2%)	(30.9%)	(48.6%)	(29.3%)
>85 Years	4	28	77	14	25
	(2.3%)	(5.0%)	(8.5%)	(9.6%)	(4.5%)
Sex					
Female	71	215	295	62	194
	(40.3%)	(38.5%)	(32.6%)	(42.5%)	(34.6%)
Male	101	308	539	79	308
	(57.4%)	(55.1%)	(59.6%)	(54.1%)	(55.0%)
NS	4	36	70	5	58
	(2.3%)	(6.4%)	(7.7%)	(3.4%)	(10.4%)
Origin					
EEA	143	213	399	118	312
	(81.3%)	(38.1%)	(44.1%)	(80.8%)	(55.7%)
Non-EEA	33	346	505	28	248
	(18.8%)	(61.9%)	(55.9%)	(19.2%)	(44.3%)
NS	0	0	0	0	0
	(0.0%)	(0.0%)	(0.0%)	(0.0%)	(0.0%)
Reporter					
HP	175	548	793	146	552
	(99.4%)	(98.0%)	(87.7%)	(100.0%)	(98.6%)
Non-HP	1	11	111	0	8
	(0.6%)	(2.0%)	(12.3%)	(0.0%)	(1.4%)
NS	0	0	0	0	0
	(0.0%)	(0.0%)	(0.0%)	(0.0%)	(0.0%)

**Table 2:** Cephalosporins included in the Watch group.

Generation	Cephalosporin
Fourth-generation cephalosporins	Cefepime
	Cefpirome
Third-generation cephalosporins	Cefcapene pivoxil
	Cefdinir
	Cefditoren pivoxil
	Cefixime
	Cefodizime
	Cefoperazone
	Cefotaxime
	Cefpodoxime proxetil
	Ceftazidime
	Ceftibuten
	Ceftizoxime
	Ceftriaxone

## Data Availability

The original contributions presented in this study are included in the article/Supplementary Materials. Further inquiries can be directed to the corresponding authors.

## Supplementary Materials

The following supporting information can be downloaded at: https://www.mdpi.com/article/10.3390/ph19010155/s1, Table S1: Frequencies of ADRs by SOCs; Table S2: Preferred terms used for reporting drug ineffectiveness, drug resistance, and off-label use; Table S3: Formulas used for the disproportionality analysis.
