# Supplementary material for: Resistance, Ineffectiveness, and Off-Label Use Related to Cephalosporins from the Reserve Group—A Pharmacovigilance Signal Detection Study on EudraVigilance Database
Source: Pharmaceuticals (Basel). 2026 Jan 15;19(1):155. doi: 10.3390/ph19010155 (PMC12844660; doi:10.3390/ph19010155)
Supplement: Supplementary file 1 [file pharmaceuticals-19-00155-s001.zip › pharmaceuticals-4071969-supplementary.pdf]

Table S1. Frequencies of ADRs by SOCs.

	Cefiderocol	Ceftaroline	Ceftazidime /avibactam	Ceftobiprole	Ceftolozane /tazobactam
Blood and lymphatic system disorders	9.7%	38.3%	10.5%	16.4%	10.7%
Cardiac disorders	2.3%	3.4%	3.5%	2.7%	4.3%
Congenital, familial and genetic disorders	0.0%	0.4%	0.2%	0.0%	0.5%
Ear and labyrinth disorders	0.6%	0.5%	0.7%	0.0%	0.2%
Endocrine disorders	0.0%	0.0%	0.0%	0.0%	0.0%
Eye disorders	0.0%	1.1%	1.4%	0.0%	0.5%
Gastrointestinal disorders	8.0%	5.7%	7.5%	7.5%	5.5%
General disorders and administration site conditions	38.1%	28.6%	26.1%	18.5%	39.1%
Hepatobiliary disorders	10.2%	2.5%	8.1%	3.4%	3.8%
Immune system disorders	2.3%	3.6%	1.8%	4.1%	2.0%
Infections and infestations	23.9%	15.2%	17.5%	5.5%	26.3%
Injury, poisoning and procedural complications	14.2%	24.0%	16.3%	23.3%	35.2%
Investigations	6.8%	14.0%	22.0%	5.5%	11.8%
Metabolism and nutrition disorders	2.3%	3.9%	2.5%	2.7%	4.6%
Musculoskeletal and connective tissue disorders	1.1%	2.7%	1.3%	0.0%	2.1%
Neoplasms benign, malignant and unspecified (incl cysts and polyps)	0.6%	0.7%	1.1%	0.7%	1.1%
Nervous system disorders	13.6%	7.2%	14.8%	37.0%	8.6%
Pregnancy, puerperium and perinatal conditions	0.0%	0.7%	0.2%	0.0%	0.0%
Product issues	0.0%	0.2%	0.2%	0.7%	1.8%
Psychiatric disorders	1.7%	2.0%	4.2%	2.7%	1.1%
Renal and urinary disorders	15.3%	7.7%	11.1%	4.8%	10.7%
Reproductive system and breast disorders	0.0%	0.2%	0.0%	0.0%	0.0%
Respiratory, thoracic and mediastinal disorders	5.7%	9.3%	4.1%	5.5%	6.1%
Skin and subcutaneous tissue disorders	9.1%	19.3%	14.9%	26.0%	8.2%
Social circumstances	0.0%	0.0%	0.0%	0.0%	0.2%
Surgical and medical procedures	0.6%	0.0%	0.7%	0.0%	3.9%
Vascular disorders	2.3%	2.1%	2.3%	3.4%	3.8%

Table S2. Preferred terms used for reporting drug ineffectiveness, drug resistance and off-label use.

Medical condition	Preferred terms
Drug resistance	Drug resistance
	Multiple-drug resistance
	Pathogen resistance
Drug ineffectiveness	Drug ineffective
	Drug effect less than expected
	Therapeutic product effect decreased
	Therapeutic product effect incomplete
	Therapeutic product ineffective
	Therapeutic response decreased
	Therapeutic response shortened
	Therapy non-responder
	Therapy partial responder
	Treatment failure
	Decreased activity
Off-label use	Contraindicated product administered
	Contraindicated product prescribed
	Off-label use
	Off-label use of device
	Product use in unapproved therapeutic environment
	Product use in unapproved indication
	Product used for unknown indication
	Unintentional use for unapproved indication

Table S3. Formulas used for the disproportionality analysis

Formula	Description
$ROR = \frac{a\ x\ d}{b\ x\ c}$	ROR = Reporting Odds Ratio<br>a = evaluated ADR for targeted drug<br>b = other ADRs for targeted drug<br>c = evaluated ADR for the drug used for comparison<br>d = other ADRs for the drug used for comparison<br>CI = confidence interval<br>SE = standard error
95% CI = exp (ln (ROR) – 1.96 x SE{ln(ROR)}) to exp (ln(ROR) + 1.96 x SE{ln(ROR)})	
$SE\{ln(ROR)\} = \sqrt{\frac{1}{a}+\frac{1}{b}+\frac{1}{c}+\frac{1}{d}}$	
